# The Quantitative Detection of Urogenital Mycoplasmas in Men with Urolithiasis

**DOI:** 10.3390/pathogens14070670

**Published:** 2025-07-08

**Authors:** Dominika Smolec, Małgorzata Aptekorz, Łukasz Filipczyk, Zygmunt Gofron, Jacek Zostawa, Robert Smolec, Tomasz J. Wąsik, Alicja Ekiel

**Affiliations:** 1 Department of Medical Microbiology, Faculty of Medical Sciences in Katowice, Medical University of Silesia, 40-752 Katowice, Poland; maptekorz@sum.edu.pl (M.A.); twasik@sum.edu.pl (T.J.W.); aekiel@sum.edu.pl (A.E.); 2 Department of Histology, Faculty of Medical Sciences in Katowice, Medical University of Silesia, 40-752 Katowice, Poland; lfilipczyk@sum.edu.pl; 3 Med Holding Emil Michalowski Specialist Hospital, 40-073 Katowice, Poland; zygmuntgofron@gmail.com; 4 Department of Urology, Faculty of Medical Sciences in Zabrze, Medical University of Silesia, 41-800 Zabrze, Poland; zostawajacek@gmail.com; 5 Department of Thoracic Surgery, Faculty of Medical Sciences in Zabrze, Medical University of Silesia, 41-800 Zabrze, Poland; robertsmolec@gmail.com

**Keywords:** qPCR, *Urogenital mycoplasmas*, urolithiasis

## Abstract

Urease-positive urogenital mycoplasmas are considered to be responsible for the formation of urinary stones. They are usually a part of the normal flora in the human urogenital tract, causing asymptomatic infections. However, many symptomatic infections with these bacteria have been reported. *M. genitalium* is recognized as a cause of male urethritis and other common genitourinary diseases. The role of other urogenital mycoplasmas is still unclear. The aim of this study was to estimate the quantitative prevalence of *Ureaplasma* spp., *M. genitalium* and *M. hominis* in men with urolithiasis using quantitative real-time PCR (qPCR). The study group comprised 100 men with urolithiasis. A total of 60 men were included in the control group. Urogenital mycoplasma DNA in urine samples was detected significantly more often among men with urolithiasis than in healthy subjects—43.0% vs. 26.6%, *p* = 0.0382, respectively. The majority of positive results (38/43) concerned *U. parvum* species, the frequency of which was higher in the study group (38.0% (38/100)) than in the control group (23.3% (14/60)), *p* = 0.0552. The median concentration of *U. urealyticum* DNA was higher in the study group compared with the control, *p* = 0.5714. However, further studies are needed to confirm the usefulness of quantitative studies in determining the role of urogenital mycoplasmas in pathology.

## 1. Introduction

Urolithiasis is an important and common health problem in the human population worldwide [1,2]. According to current data, urolithiasis affects both women and men on a comparable scale [3]. Infection with bacteria that synthesize urease may lead to the formation of infection stones by breaking down urea into ammonia and carbonate. An increase in alkaline pH and ammonia is associated with the formation of struvite stones, which are composed of magnesium ammonium phosphate. The most important urease-producing bacteria causing urinary tract infections are *Proteus, Providencia* and *Morganella* [4,5]. *Ureaplasma* spp. strains also produce urease. However, routine urine culture procedures do not include fastidious and slow-growing bacteria [5]. Urogenital mycoplasmas have a tendency to colonize the mucous membranes of the human urogenital tract as commensal inhabitants [6]. These bacteria are associated with NGU (Nongonococcal Urethritis), NCNGU (Nonchlamydial Nongonococcal Urethritis) cervicitis, PID (Pelvic Inflammatory Disease), BV (Bacterial Vaginosis), pathological course of pregnancy, premature birth and infections in newborns, although their role is still discussed [7,8,9,10]. *M. genitalium* is the only mycoplasma with clearly defined pathogenicity [10]. *Ureaplasma* spp. are the most prevalent mycoplasma species isolated from the urogenital tract [11]. Colonization by mycoplasmas poses a risk of infection for patients with immunodeficiencies. Moreover, reports on opportunistic infections located outside of the urogenital tract caused by *M. fermentans, M. pirum* and *M. penetrans* indicate the importance of colonization with these bacteria [9,12,13]. Conventional polymerase chain reaction (PCR) and real-time PCR have been used for the detection and identification of urogenital mycoplasmas, including those that are rarely included in routine diagnostic procedures. However, the quantitative real-time PCR (qPCR) method is worth considering for its high sensitivity in detection and for explaining the importance of these organisms in pathology.

The formation of deposits in the urinary tract is favored by urinary stasis and urinary tract infections caused by urease-producing bacteria. Therefore, the main aim of this study was to assess the prevalence of urogenital mycoplasmas in patients with urolithiasis and in a control group using the quantitative real-time PCR method.

## 2. Materials and Methods

### 2.1. Study Participants

This study group comprised 100 men with urolithiasis (median age 57, range 23–81) and 60 healthy men without urinary tract infections as the control group (median age 47, range 21–68), *p* = 0.0576. Basic laboratory tests, including morphology, urinalysis and urine culture, were performed in all patients. Patients and the control group were not undergoing antibiotic, chemotherapy and antifungal therapy; STIs were not diagnosed; and they had not undergone any medical procedures for at least 4 weeks prior to this study. All study participants gave written consent and were informed on how to properly collect urine samples for testing.

### 2.2. Pretreatment of Clinical Specimens and DNA Extraction

Samples of morning first void urine (FVU) (5–10 mL) were collected in sterile plastic containers and transported to the laboratory at +4 °C. The FVU samples (approximately 1 mL) were centrifuged at 15,000× *g* for 30 min. The supernatant was removed and the pellet was re-suspended in 1 mL of Lyse BG buffer. Genomic DNA was extracted from the pretreated specimens using the Gene MATRIX Bacterial and Yeast Genomic DNA Purification Kit (EURx, Gdańsk, Poland), according to the manufacturer’s protocol. The internal control (IC) was added to each sample as a control to monitor the extraction procedure and detect possible reaction inhibitors.

### 2.3. qPCR Assays

The AmpliSens Florocenosis/Mycoplasma-FRT real-time PCR test (AmpliSens, Prague, Czech Republic) was used to detect *U. parvum*, *U. urealyticum* and *M. hominis* DNA in 160 FVU samples. The quantitative detection of DNA was based on the linear relationship observed when the fluorescent signal begins to increase exponentially (cycle threshold, Ct). For quantitative detection, the DNA of clinical samples was amplified simultaneously with DNA calibrators (samples with a known concentration of target DNA). The results of DNA calibrator amplification were used to construct a calibration curve and calculate the target DNA concentration in the samples. In the AmpliSens test, the concentration of DNA was determined by the number of genome equivalents of microorganism cells per ml of the clinical sample (GE/mL). These obtained values reflect the absolute concentration of microorganisms in the clinical material. The results of amplification were recorded in four different fluorescence channels (Table 1). The test was performed in accordance with the manufacturer’s instructions using the real-time PCR instrument LightCycler 96 (Roche, Basel, Switzerland).

### 2.4. Qualitative Real-Time PCR

The AmpliSens *Mycoplasma genitalium* FRT PCR test (AmpliSens, Czech Republic) was used for *M. genitalium* DNA detection (targeting the *gyrB* gene) in accordance with the manufacturer’s instructions. PCR was performed using the real-time PCR instrument LightCycler 96 (Roche).

### 2.5. Conventional PCR

*M. fermentans* and *M. pirum* DNA identification was performed by PCR using specific primers (Table 2) and a Taq PCR Core Kit (Qiagen Inc., Hilden, Germany) in Mastercycler thermocycler (Eppendorf AG, Hamburg, Germany), under the following conditions: *M. pirum*: 94 °C for 2 min, 35 × (94 °C for 30 s, 55 °C for 30 s, 72 °C for 1 min), 72 °C for 5 min; *M. fermentans*: 94 °C for 2 min, 35 × (94 °C for 30 s, 55 °C for 45 s, 72 °C for 50 s), 72 °C for 5 min. Negative samples were checked for the presence of amplification inhibitors by PCR reactions using beta-globin control primers. Amplified products were visualized under UV light after electrophoresis in 2% agarose gel containing ethidium bromide and recorded using the GeneSys image archiving and analysis system (Syngene, Cambridge, UK). Reference strains *M. fermentans* ATCC 199989D and *M. pirum* ATCC 25960D were used as positive controls.

### 2.6. Statistical Analysis

Statistical analysis was performed using Statistica, version 13 (TIBCO Software INC. 2017, https://www.tibco.com/). Differences between groups were analyzed using the chi-square test (χ2) and Fisher’s exact test. The Shapiro–Wilk test was used to assess the normality of distribution. The Mann–Whitney U test was used to compare medians between groups. A significance level of *p* < 0.05 was considered statistically significant.

## 3. Results

Urogenital mycoplasma strains were detected significantly more often in the study group than in the control group (43.0% vs. 26.6%, respectively; *p* = 0.0382) (Table 3). *U. parvum* DNA was more frequently found in patients with urolithiasis (38/100, 38.0%) than in healthy men (14/60, 23.3%), *p* = 0.0552. The DNA of *U. parvum* was detected more often than that of *U. urealyticum* in study group (38% vs. 5%, respectively; *p* < 0.0005). *U. parvum* was also the most frequent of all mycoplasmas in both groups: patients (38/100 vs. 7/100; *p* < 0.0005) and controls (14/60 vs. 3/60; *p* = 0.0041). The presence of *M. genitalium* and *M. pirum* DNA was not detected in either group.

In all samples with detected urogenital mycoplasma, the concentration of DNA ranged from >10^2^ to 10^4^ GE/mL. Among the study group, urogenital mycoplasma DNA was detected significantly more often in the lower range (>10^2^–10^3^ GE/mL; 29/100) than in higher range (>10^3^–10^4^ GE/mL; 15/100), *p* = 0.0253. Similarly, in the control group, samples with urogenital mycoplasma DNA in the range >10^2^–10^3^ GE/mL (11/60) were more frequently detected than those in the range >10^3^–10^4^ GE/mL (6/60), *p* = 0.0381 (Table 4).

In the study group and control group, the median concentration of urogenital mycoplasma DNA showed no statistically significant difference (7.8 × 10^2^ GE/mL and 6.6 × 10^2^ GE/mL, respectively). No significant difference was found in the median *U. parvum* DNA concentration between the two groups (7.7 × 10^2^ GE/mL in the study group vs. 6.6 × 10^2^ GE/mL in the control group). The concentration of U. *urealyticum* DNA was higher in the study group (median = 1.1 × 10^3^ GE/mL) compared with the control group (median = 6.7 × 10^2^ GE/mL), *p* = 0.5714.

The results of urogenital mycoplasma DNA detection in relation to selected blood and urine test parameters in men from the study group were also analyzed (Table 5).

In all samples from patients with a negative urine culture, urogenital mycoplasma DNA was detected significantly more often in patients with leukocyturia compared to those without leukocyturia (29/43 vs. 15/54, respectively; *p* = 0.0002) (Table 6).

Leukocyturia was more frequently detected in samples with a higher concentration of urogenital mycoplasma DNA (>10^3^–10^4^ GE/mL; 18/43). No significant differences were found in relation to the other parameters.

## 4. Discussion

Urolithiasis is one of the most common urological diseases, with an increasing tendency [16,17,18]. Ureaplasma species produce urease, an important factor in the formation of urinary tract stones. Struvite stones are a type of urinary and kidney stone that form almost exclusively in patients infected with urease-positive bacteria. The role of *Ureaplasma* spp. strains in the stone formation process has been demonstrated by many authors [19,20]. In our study, urogenital mycoplasmas were detected by qPCR in 43% of 100 men with urolithiasis. This was significantly more frequent compared to the controls (*p* = 0.0382). Mobarak and Tharwat showed the presence of *Ureaplasma* spp. in the mid-stream urine of 26.7% of men with urolithiasis using the culture method [21]. Using the same method, a higher percentage (62.5%) of urogenital mycoplasmas was detected in patients with urolithiasis, as shown by Shafi et al., but in that study, urine was collected from catheters after nephrolithotomy [22]. The group of men most frequently tested for urogenital mycoplasmas were patients with urethritis, especially NGU. In this group of patients, Maeda et al. showed the prevalence of *U. urealyticum* and *U. parvum* in 16.3% and 7.8% of men with NGU, respectively. In an even higher percentage of NCNGU patients, *U. urealyticum* was detected in 18.8% and *U. parvum* in 8.8% [23]. In our study group, *U. parvum* was more frequently detected than *U. urealyticum* (38% vs. 5%, respectively; *p* < 0.0005), but our study consisted of patients with urolithiasis. Frølund et al. showed *U. urealyticum* dominance in patients with acute NGU, whereas *U. parvum* was dominant in patients with chronic NGU and in the control group [24]. In the development of urolithiasis, both species may be important because both produce urease.

In our research, we also included *M. genitalium* detection, which is the only mycoplasma with a clearly established role in the etiology of urogenital infections [8,25]. In our study, there were no positive results for *M. genitalium*; however, taking into account the published results of works by other authors, this species mainly occurs in patients with clinical symptoms of urogenital tract infection [8,26]. Yoshida et al. also did not demonstrate the presence of *M. genitalium* in the FVU of asymptomatic patients [27]. Strains of this species are rare. Screening studies from the United States, UK, Denmark and Australia have confirmed *M. genitalium* infection in 1–3% of men and women [28,29,30,31]. The frequency of DNA detection for this species increases significantly in sexually transmitted disease clinic patients. Among men treated at the STI clinic in Seattle (USA), the percentage of people testing positive for this pathogen was 9% [32]. In another study from France, in young men with symptoms of urethritis, the presence of *M. genitalium* DNA was detected in 21.7%. In the same study, in men without urinary tract infections, *M. genitalium* DNA was not detected [33].

Many authors emphasize that *M. fermentans, M. pirum* and *M. penetrans* may be etiological agents of opportunistic infections in at-risk patient groups, especially in immunocompromised individuals. Preiswerk et al. drew attention to the possibility of *M. penetrans* infections occurring in immunocompromised patients, describing the case of a 38-year-old woman after a kidney transplant who was diagnosed with bacteremia of this etiology [34]. Nevertheless, the occurrence of *M. fermentans, M. penetrans* and *M. pirum* species and their pathogenic potential have not been sufficiently examined or confirmed, and the reported cases still mainly concern patients infected with HIV. The presence of *M. fermentans* was confirmed in the kidney tissue of patients with nephropathy and in the bronchopulmonary lavage of AIDS patients with diagnosed pneumonia who developed respiratory distress syndrome [35,36]. In a different study among women (pregnant and infertile) without pathology, HIV infection or nephropathy, the prevalence of *Mycoplasma fermentans* was noted in 14.1% of these patients. Furthermore, *M. fermentans* has been found to be associated with bacterial vaginosis [37]. This may indicate that *M. fermentans* may be involved in changes to the vaginal microbiome, and research into these rarely detected microorganisms should be continued.

### 4.1. Quantification of Urogenital Mycoplasmas in Patients with Urolithiasis and in the Control Group

*U. urealyticum* strains are more often indicated as the cause of symptomatic infections, mainly in the case of NGU in men. In our study, the concentration of *U. urealyticum* DNA was higher in patients (median = 1.0 × 10^3^ GE/mL) than in the controls (median = 6.7 × 10^2^ GE/mL). The concentration of tested DNA in our study group was similar to the group with acute NGU in the study conducted by Frølund et al. They showed that the bacterial load of *U. urealyticum* was higher in both acute NGU (median = 6.7 × 10^3^ GE/mL) and chronic NGU (median = 5.5 × 10^4^ GE/mL) than in the control group (median = 313 GE/mL) (*p* = 0.002 and *p* = 0.02, respectively). The bacterial load of *U. parvum* was similar in the NGU groups compared to the control group [24]. The concentration of *U. parvum* DNA in our study and the control group did not show a statistically significant difference. Strauss et al. showed that a strong positive result (by semi-quantitative PCR test in urine samples) for *U. parvum* occured significantly more often in symptomatic patients compared to asymptomatic patients. However, these strongly positive samples, examined by qPCR, exceeded 7.5 × 10^5^ GE/mL [38]. *U. parvum* at lower concentrations of GE/mL were often found in subjects without symptoms of infection. Deguchi et al. reported that the bacterial load of *U. parvum* in the male urethra was less than 5 × 10^3^ cells of *U. parvum*/mL in most cases (83%). The authors concluded that *U. parvum* typically colonizes the male urethra, but in cases with high levels of these strains, an inflammatory response may occur [39].

### 4.2. The Importance of Leukocyturia Among Urogenital Mycoplasma Infections

Leukocyturia is present in both infectious and non-infectious urinary tract diseases. Wanic-Kossowska et al. pointed out that in the case of leukocyturia and a negative urine culture test, the infection may be caused by *Mycoplasma* spp. and/or *U. urealyticum* [40]. In our study, leukocyturia was found significantly more often in patients with a positive test result for urogenital mycoplasmas compared to a negative result. Deguchi et al. showed that the presence of *U. parvum* DNA at a load of ≥5  ×  10^3^ cells/mL in FVU of the studied men was correlated with the presence of leukocyturia (>12.5 leukocytes/µL in FVU) [38]. In our study, leukocyturia was more often detected in patients with a higher concentration of urogenital mycoplasma DNA (>10^3^–10^4^ GE/mL). Dupin et al. found *M. genitalium* in 21.7% of symptomatic patients with urethritis and leukocyturia, whereas these bacteria have not been detected in patients without leukocyturia [33]. Vlasic-Matas et al. found that in patients with recurrent urinary tract infections, leukocyturia was more commonly associated with *U. urealyticum* than with *C. trachomatis* infection [41]. The study by Park and Lee also showed an association between the prevalence of *U. urealyticum* and an increased number of leukocytes in urine (>5 WBC) [42]. The results of our study, along with those of previous studies, showed a significant association between leukocyturia and the presence of urogenital mycoplasmas. This confirms that patients with symptoms of urogenital infection and a negative urine culture test (for pathogens in routine urine testing) should be tested for atypical pathogens, such as urogenital mycoplasmas.

## 5. Conclusions

In our study, we found that urogenital mycoplasma DNA among men with urolithiasis occurred more frequently than in the control group. The concentration of *U. urealyticum* DNA was higher in the study group compared with the control group; however, further studies are needed to confirm the usefulness of quantitative studies in determining the role of urogenital mycoplasmas in pathology. This study also showed the association between leukocyturia and the presence of urogenital mycoplasmas in the examined patients. Therefore, in cases where determining the etiological agent of urogenital infection is difficult in symptomatic patients with confirmed leukocyturia, diagnostic testing for *M. hominis, U. urealyticum* and *U. parvum* should be recommended.

## Figures and Tables

**Table 1:** Fluorescence channels and target genes for the detection of urogenital mycoplasma DNA.

Channel for Fluorophore	FAM	JOE	ROX	Cy5
DNA target	*U. parvum*	*U. urealyticum*	*M. hominis*	Internal Control-FL (IC) DNA
Target gene	Urease gene	Urease gene	16S rRNA	β-globin gene

**Table 2:** Primers, nucleotide sequences and expected PCR product sizes for the detection of *M. pirum* and *M. fermentans* DNA.

Starters	5′-3′ Nucleotide Sequences	Product Size (bp)	Gene	Reference
*M. pirum* primer 7 primer 8	5′-ATA CAT GCA AGT CGA TCG GA-3′ 5′–ACC CTC ATC CTA TAG CGG TC-3′	180	16S rRNA	[14,15]
*M. fermentans* RW004 RW005	5′-GGA CTA TTG TCT AAA CAA TTT CCC-3′ 5′-GGT TAT TCG ATT TCT AAA TCG CCT-3′	206	Element IS-like	

**Table 3:** The prevalence of urogenital mycoplasma in men with and without urolithiasis.

	Patients with Urolithiasis [*n* = 100]	Control Group [*n* = 60]	*p*
	Number (%)		
*U. parvum*	38 (36.0)	14 (23.3)	0.0552
*U. urealyticum*	5 (5.0)	2 (3.3)	0.6189
*M. hominis* *	1 *	1 *	-
*M. fermentans* *	1 *	0	-
Total samples with urogenital mycoplasma DNA	43 (43.0)	16 (26.6)	0.0382

* detected in the sample with *U. parvum.*

**Table 4:** Genome equivalents of the urogenital mycoplasma DNA identified in urine from patients with urolithiasis and the control group.

	Patients with Urolithiasis [*n* = 100]	Control Group [*n* = 60]	*p*
	The number of samples with DNA concentration		
	in the range >10^2^–10^3^ GE/mL		
*U. parvum*	26	9	0.1032
*U. urealyticum*	2	1	0.8808
*M. hominis*	1	1	0.7141
Total	29 ^#^	11 ^##^	0.1029
	in the range >10^3^–10^4^ GE/mL		
*U. parvum*	12	5	0.4676
*U. urealyticum*	3	1	0.6021
Total	15 ^#^	6 ^##^	0.3660

^#^ The number of samples with DNA concentration in the range >10^2^–10^3^ GE/mL (29/100) vs. the number of samples in the range >10^3^–10^4^ GE/mL (15/100) in the study group (*p* = 0.0253). ^##^ The number of samples with DNA concentration in the range >10^2^–10^3^ GE/mL (11/60) vs. the number of samples in the range >10^3^–10^4^ GE/mL (6/60) in the control group (*p* = 0.0381).

**Table 5:** The prevalence of urogenital mycoplasma DNA in relation to selected laboratory parameters in patients with urolithiasis, *n* = 100.

Selected Laboratory Parameters	The Number of Patients (the Number of Patients with Urogenital Mycoplasma DNA)
Urine	
Leukocyturia > 5 WBC/FOV	47 (29)
RBC > 5 RBC/µL	34 (15)
Urine culture test negative	97 (43)
Blood	
Leukocytosis N: >11,000/µL	9 (0)
Creatinine > 0.9 mg/dL	10 (1)

**Table 6:** The prevalence of urogenital mycoplasma DNA in the urine of patients with and without leukocyturia, *n* = 97 *.

	Men with Urolithiasis		
	Without Leukocyturia	with Leukocyturia	*p*
With mycoplasma DNA detected	43	29	0.0002
With no mycoplasma DNA detected	54	15	
Total	97 *	44	

* The results of three samples with leukocyturia and a positive urine culture were not included in this analysis.

## Data Availability

The data presented in this study are available on request from the corresponding author.
